# Supplementary material for: Choosing panels of genomics assays using submodular optimization
Source: Genome Biol. 2016 Nov 15;17:229. doi: 10.1186/s13059-016-1089-7 (PMC5111315; doi:10.1186/s13059-016-1089-7)
Supplement: Additional file 1 — Additional figures and text. (PDF 606 kb) [file 13059_2016_1089_MOESM1_ESM.pdf]

Supplement to "Choosing panels of genomics assays using submodular optimization "

Kai Wei*[1], Maxwell W. Libbrecht*[2], Jeffrey A. Bilmes[1,2], and William Stafford Noble[3,2]

[1]Department of Electrical Engineering, University of Washington
[2]Department of Computer Science and Engineering, University of Washington
[3]Department of Genome Sciences, University of Washington

August 30, 2016

*Contributed equally

1 Supplementary Note 1: Comparison of potential objective functions

We compared four classes of submodular objectives for selecting panels of assays. All functions are defined via a pairwise similarity graph, where we denote $r_{v,v'}$ as the similarity measure between the v and $v' \in V$.

Facility location function:

$$f_{\text{fac}}(A) = \sum_{v \in V} \max_{a \in A} r_{v,a} \tag{1}$$

This function is monotone and submodular.

Saturated coverage function:

$$f_{\text{sat}}(A) = \sum_{v \in V} \min\{\sum_{a \in A} r_{v,a}, \beta \sum_{v' \in V} r_{v',v}\}, \tag{2}$$

In this function, $0 \leq \beta \leq 1$ is a hyperparameter that controls the saturation of the coverage for each item $v \in V$. In the experiment we set $\beta = k/n$, where k is the target number of assays to select, and n is the size of entire set V of all available assays. The saturated coverage function is also monotone submodular.

Diversity function:

$$f_{\text{div}}(A) = -\sum_{a \in A} \sum_{a' \in A} r_{a,a'} \tag{3}$$

The diversity function evaluates a subset A as the sum of the pairwise dissimilarity among all items in the subset. Maximizing this function naturally encourages choosing a diverse set of items. The diversity function is submodular, but not monotone.

Log determinant function:

$$f_{\text{log-det}}(A) = \log\det(I + \lambda S_A) \tag{4}$$

In this function, $\lambda > 0$ is a hyperparameter, and S_A is the pairwise similarity matrix indexed by the subset $A \subseteq V$. The log determinant function satisfies submodularity and monotonicity. This function has been shown to naturally capture the notion of diversity in a data set; therefore, maximizing this function always leads to a set of diverse items.

We measure the performance of these submodular objectives by examining how their objective valuations correlate with the three proposed evaluation metrics (assay imputation, functional element prediction, and annotation-based evaluation). The experiment is performed under the selection of past assays setting, where we compute the similarity between a pair of assays using the Pearson correlation. Given an evaluation metric, a cell type c, a selection budget K, and a submodular objective f, we randomly draw n subsets $\{A_i\}_{i=1}^n$ of assays from the cell type c, where each subset A_i is of size K. We then compute the Spearman correlation between the submodular valuation $\{f(A_i)\}_{i=1}^n$ and the performance measure $\{y_i\}_{i=1}^n$ under the evaluation metric. We report the correlation measure averaged over budget constraints $K = 3, 4, 5, 6$. In the experiment we set $n = 20$, and we test on the cell types K562, H1-hESC, and GM12878.

The results (Figure 1) suggest that the facility location function, in most cases, yields the highest correlation with the three evaluation metrics.

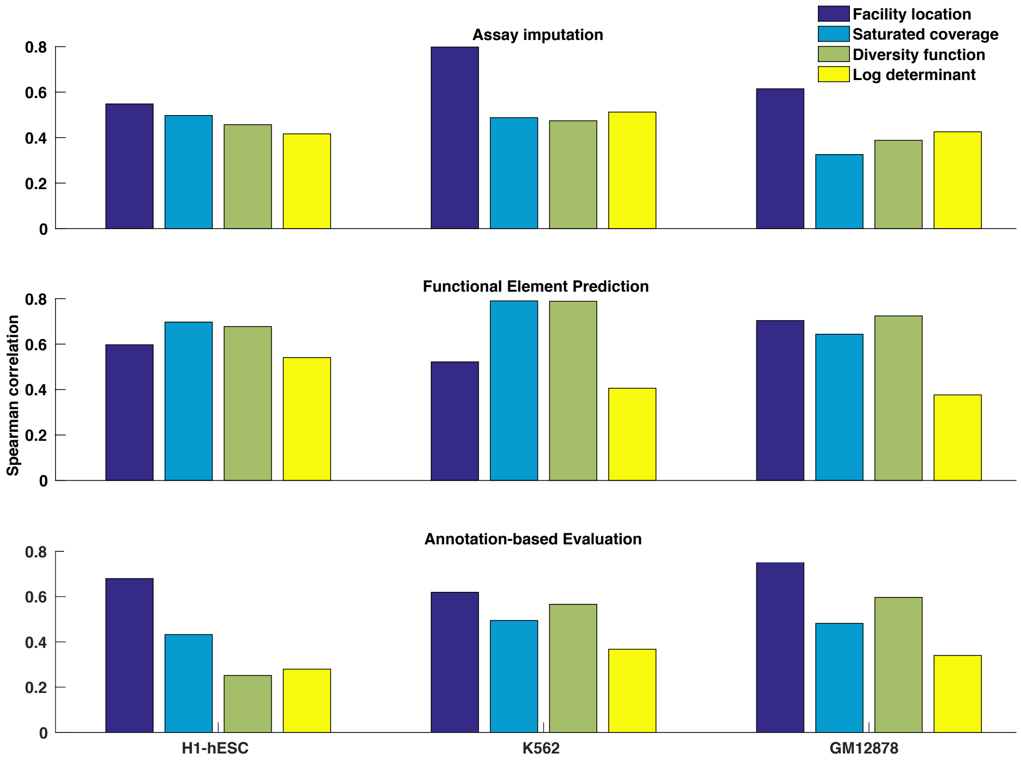

Figure 1: Comparison among various submodular functions in terms of the Spearman correlation between function valuation and the performance metrics.

2 Supplementary Note 2: Comparison among various similarity measures

In this section, we study how the performance of SSA for the selection of future assays scenario varies with different choices of similarity aggregation strategies. We focus on strategies for defining the pairwise similarity between assay types in an evaluating cell type c given the assays performed on cell types other than c. We consider the six aggregation strategies r^1, r^2, r^3, r^4, r^5, and r^6, where they take the average, 0th, 25th, 50th, 75th, and 100th percentile over the available similarity scores, respectively. For completeness, we also give their corresponding mathematical definition below:

$$r_{s_i,s_j} \triangleq \frac{1}{|\mathcal{S}^{a_i} \setminus s_i|} \frac{1}{|\mathcal{S}^{a_j} \setminus s_j|} \sum_{s \in \mathcal{S}^{a_i} \setminus s_i} \cdot \sum_{s' \in \mathcal{S}^{a_j} \setminus s_j} |\rho_{s,s'}|, \tag{5}$$

$$r^2_{s_i,s_j} \triangleq \text{percentile}(\{|\rho_{s,s'}| : s \in \mathcal{S}^{a_i}, s' \in \mathcal{S}^{a_j} \setminus s_j\}, 0), \tag{6}$$

$$r^3_{s_i,s_j} \triangleq \text{percentile}(\{|\rho_{s,s'}| : s \in \mathcal{S}^{a_i}, s' \in \mathcal{S}^{a_j} \setminus s_j\}, 25), \tag{7}$$

$$r^4_{s_i,s_j} \triangleq \text{percentile}(\{|\rho_{s,s'}| : s \in \mathcal{S}^{a_i}, s' \in \mathcal{S}^{a_j} \setminus s_j\}, 50), \tag{8}$$

$$r^5_{s_i,s_j} \triangleq \text{percentile}(\{|\rho_{s,s'}| : s \in \mathcal{S}^{a_i}, s' \in \mathcal{S}^{a_j} \setminus s_j\}, 75), \tag{9}$$

$$r^6_{s_i,s_j} \triangleq \text{percentile}(\{|\rho_{s,s'}| : s \in \mathcal{S}^{a_i}, s' \in \mathcal{S}^{a_j} \setminus s_j\}, 100), \tag{10}$$

where the function percentile(C, p) returns the pth percentile of the items in the list C sorted in non-decreasing order.

We evaluate the performance of the aggregation strategies using the facility location function. In particular we utilize the six different aggregation strategies to compute the pairwise similarity measure for defining the facility location function. We test separately on three cell types: H1-hESC, K562, and GM12878. Similar to the previous experiment for comparing among different submodular objectives, we measure the performance of an aggregation strategy as how the valuation given by facility location function defined via the similarity matrix computed using this strategy correlates with the three proposed evaluation metrics. Following the same evaluation procedure we show the Spearman correlation measure for each aggregation strategy on each cell type and evaluation metric in Figure 2. We observe that the aggregation strategies of taking the mean or 75th percentile yield the best performance for most cell types and most evaluation metrics. Between these two strategies we observe that the aggregation by mean performs marginally better. Therefore we choose it as the strategy for computing the similarity between assay types in the propose approach SSA under the selection of future assays setting.

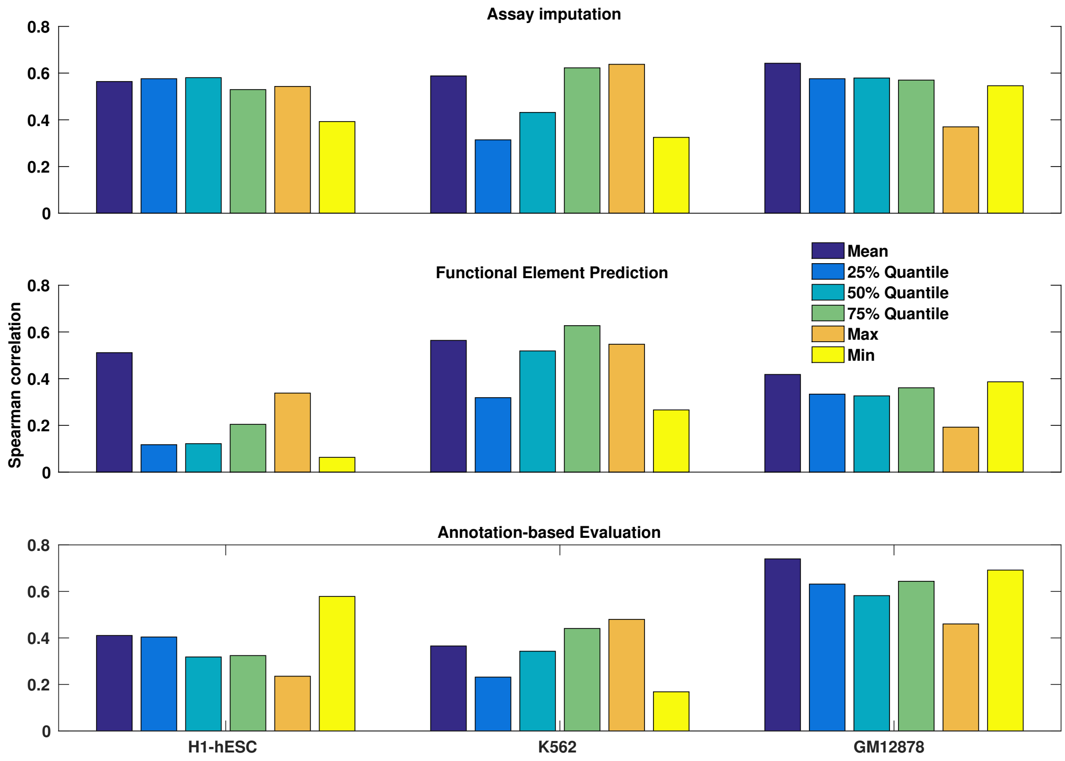

Figure 2: Comparison among various similarity aggregation strategies in terms of the Spearman correlation between the facility location function valuation instantiated by the similarity measure and the performance metrics.

3 Supplementary Note 3: Comparison of different supports for similarity computation

In this section, we examine how the similarity measure may be affected if we change the support for deriving the similarity between assay types. In the main paper, we derive the similarity between assays as the Pearson correlation between the values of these two assays on a randomly chosen subset of genomic positions. In this section, we explore deriving the similarity based only on the set of genomic positions that are identified as DNase peaks, since the response in the transcription factors is only captured on these DNase peaks positions.

In Figure 3, we report the performance of the DNase peaks based SSA on all evaluation metrics as well as the three cell types. For completeness, we also show the corresponding performance of the variant of SSA that we used in the main main paper in Figure 4. Note that this variant of SSA has the similarity measure computed over a randomly sampled subset of genomic positions. To better illustrate the comparison between these two variants, we plot the performance measure of the DNase peaks approach (each bar in Figure 3) against the random genomic positions approach (each bar in Figure 4) in the form of scatter plot (Figure 5). We observe that consistent and significant improvements over the random selection baseline are achieved by the variant of SSA using DNase peaks only. Between the two variants of SSA, it is hard to establish the superiority of one variant over the other according to Figure 5.

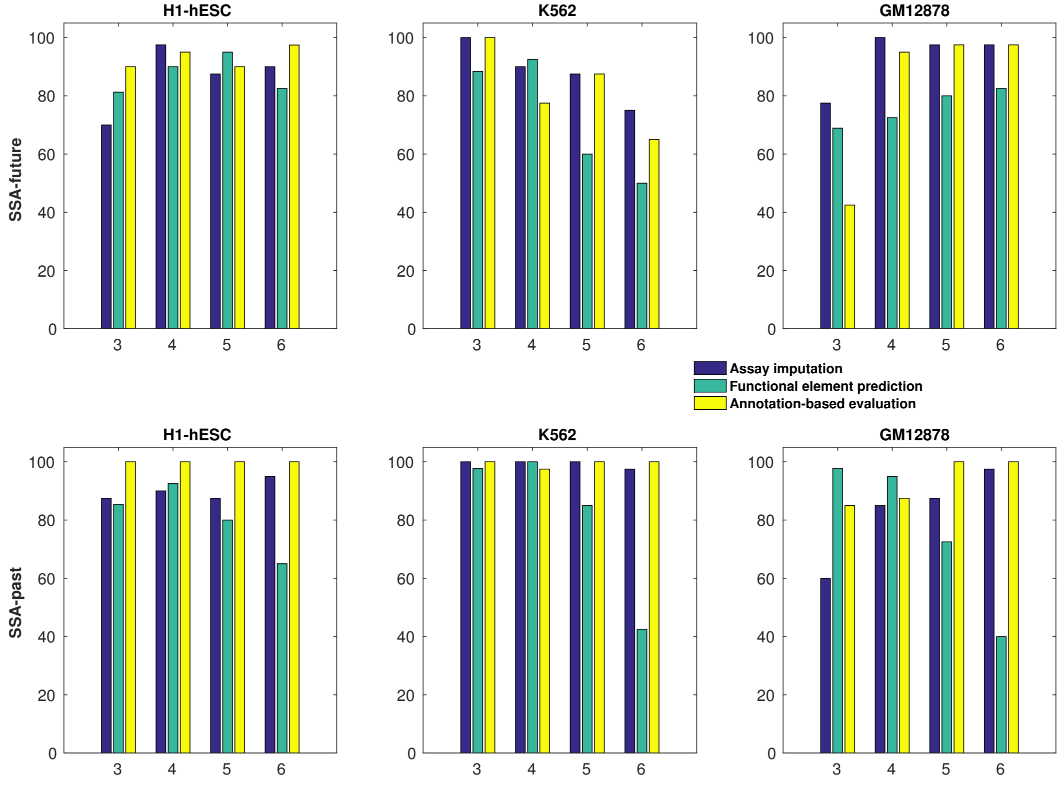

Figure 3: Performance of the Dnase peaks-based SSA relative to an estimate of the performance on all possible panels. The vertical axis shows the fraction (%) of panels that perform worse than the SSA-chosen panel for a given setting, estimated by comparing to 40 randomly-selected panels.

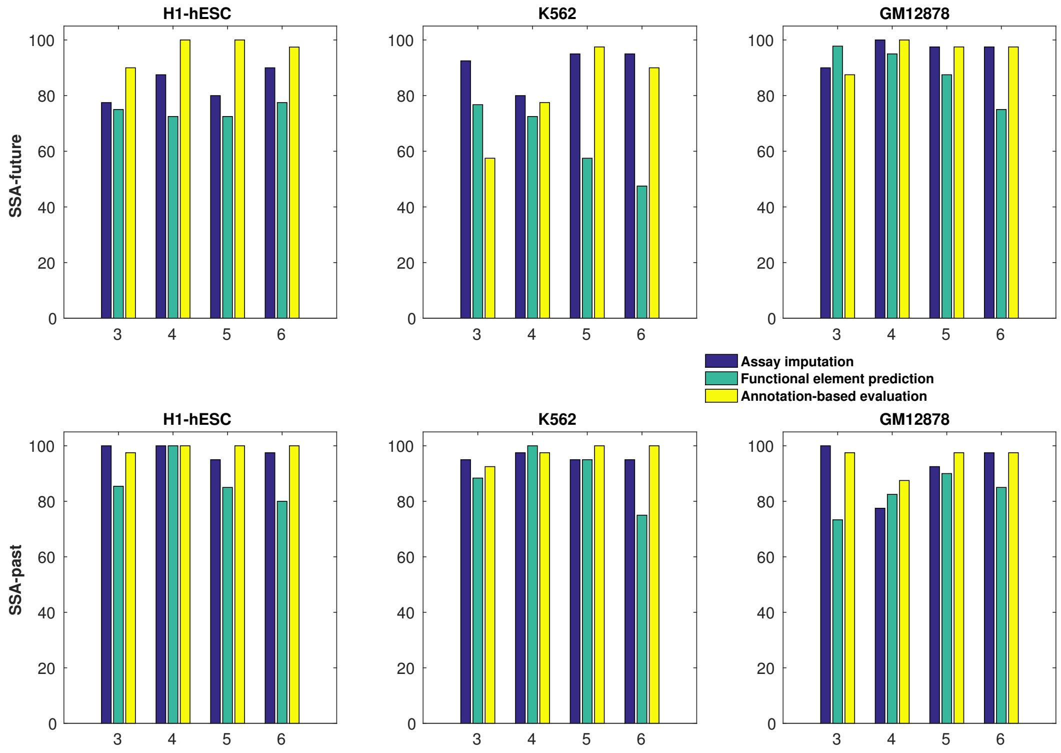

Figure 4: Performance of the random genomics positions-based SSA relative to an estimate of the performance on all possible panels.

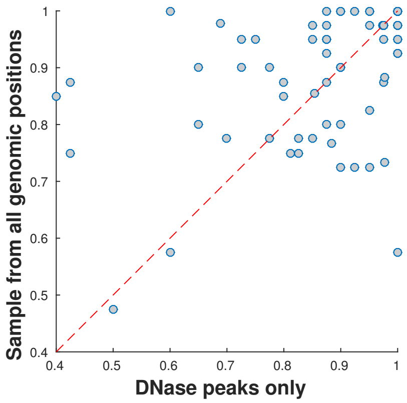

Figure 5: Scatter plot between the two variants of SSA with different genomic support for similarity computation. Each dot in the plot corresponds to the performance (again measured as relative to an estimate of the performance on all possible panels) of the two variants of SSA for a selection budget evaluated using a metric in a cell type (i.e., one bar in Figure 3 and its counterpart in Figure 5). Its x- and y-values are the performance measure for the DNase peaks-based SSA and random genomic positions-based SSA, respectively.

4 Supplementary Note 4: Comparison of different correlation metrics as the similarity measure

In this section, we examine how the similarity measure may be affected by different choice of correlation metrics. For the results reported in the main paper, we derive the similarity between assays via the absolute Pearson correlation. Here, we explore a variant of SSA whose similarity measure is defined via the absolute Spearman correlation. In Figure 6, we report the performance of such variant on all evaluation metrics as well as the three cell types. To contrast the performance difference between the Pearson correlation-based SSA and Spearman correlation-based SSA, we again show a scatter plot of the performance achieved between these two variants in Figure 7. We observe that the Pearson correlation based SSA significantly and consistently outperforms the Spearman correlation counterpart suggesting that Pearson correlation is more effective for capturing similarity between assays in our tasks.

Spearman correlation (essentially, Pearson correlation of the ranks) and also Pearson correlation on a subset of sites (e.g., the DNase hypersensitive cases, see Supplementary Note 3) constitutes a similarity measure that is non-linearly related to the global Pearson correlation. Mutual information, defined as $I(X;Y) = \int p(x,y) \log p(x,y)/p(x)p(y)dxdy$ is also strongly related to the Pearson correlation. Assuming that ρ_{xy} is the Pearson correlation and the variables are Gaussian related, then the mutual information is expressed (Kullback, 1968) as $I(X;Y) = -\frac{1}{2}\log_2(1-\rho_{xy}^2)$. Hence, the utilization of Pearson correlation is essentially a form of linear mutual information. In the non-Gaussian case, the mutual information also incorporates any non-linearly relations, but we have found in our experiments that the linear first-order relationship is often dominant. Estimating mutual information in the non-Gaussian case, however, is plagued with additional decisions regarding how a parametric or non-parametric model for $p(x,y)$ should be estimated, and how to control for bias in the estimations based on the available data used to estimate $p(x,y)$, not to mention that the computation of $p(x,y)$ can in some cases be non-trivial. Hence, for these reasons, and since it appears to perform better than the Spearman correlation, we have concentrated on Pearson correlation (e.g., linear mutual information) in our experiments in this paper. Utilizing other forms of non-linear mutual information besides Spearman correlation we leave to future work.

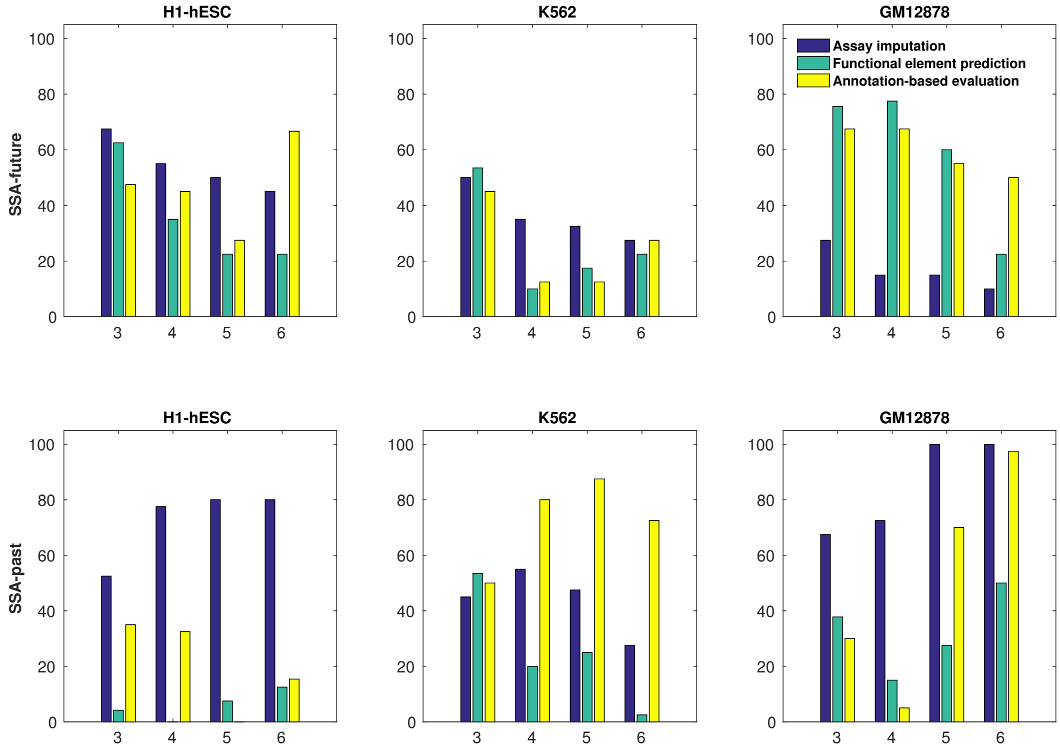

Figure 6: Performance of the absolute Spearman correlation-based SSA relative to an estimate of the performance on all possible panels.

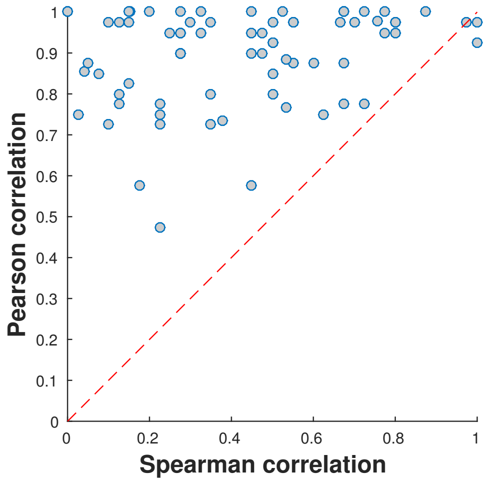

Figure 7: Scatter plot between the two variants of SSA with different correlation metrics as the similarity measure. Each dot in the plot corresponds to the performance (again measured as relative to an estimate of the performance on all possible panels) of the two variants of SSA for a selection budget evaluated using a metric in a cell type (i.e., one bar in Figure 6 and its counterpart in Figure 5). Its x- and y-values are the performance measure for the Spearman correlation-based SSA and the Pearson correlation-based SSA, respectively.

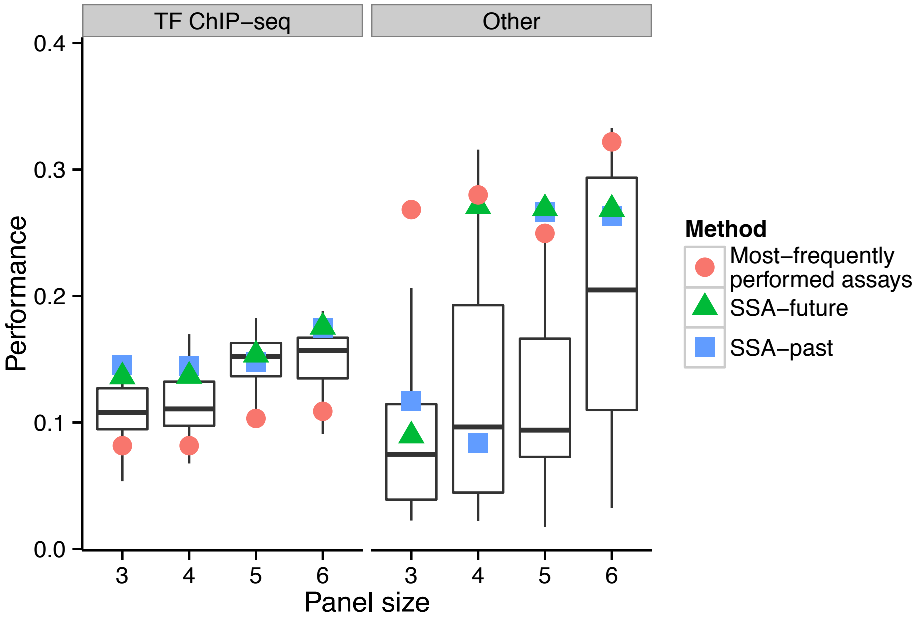

Figure 8: Same as the "Assay Imputation" panel of Figure 5B, but split according to the evaluation target. The left panel shows assay imputation performance where the target is a transcription factor ChIP-seq assay; the right panel shows performance for all other assay types.

Supplementary Tables

**Choice order**	**Assay type**	**Singleton score**	**Objective gain**
1	NFATC1	36.38	36.38
2	ILF2	33.37	4.60
3	PHF8	27.01	3.06
4	POU5F1	22.36	2.26
5	SMARCC1	13.63	1.93
6	IRF4	28.22	1.42
7	HNF4G	15.78	1.33
8	HMGN3	24.93	1.27
9	ILF3	29.09	1.04
10	ZNF217	16.00	1.03

Table 1: The choice order of SSA on all assay types.

References

Kullback S. 1968. *Information Theory And Statistics.* Dover.

Order	None	H3K4me1	H3K4me2	H3K9ac	H2A.Z	H4K20me1	H3K27ac
1	H3K4me3	H3K4me1	H3K4me2	H3K9ac	H2A.Z	H4K20me1	H3K27ac
2	H3K79me2	H3K4me3	H3K79me2	H3K79me2	H3K79me2	H3K4me3	H3K79me2
3	H3K9me3	H3K79me2	H3K4me3	H3K9me3	H3K4me3	H3K79me2	H3K4me3
4	H3K27me3	H3K9me3	H3K9me3	H3K27me3	H3K9me3	H3K9me3	H3K9me3
5	H3K36me3	H3K27me3	H3K27me3	H3K4me3	H3K36me3	H3K27me3	H3K27me3
6	H3K4me1	H3K36me3	H3K36me3	H3K36me3	H3K27me3	H3K36me3	H3K36me3
7	H3K4me2	H3K4me2	H3K4me1	H3K4me1	H3K4me1	H3K4me1	H3K4me1
8	H3K9ac	H3K9ac	H3K9ac	H3K4me2	H3K4me2	H3K4me2	H3K4me2
9	H2A.Z	H2A.Z	H2A.Z	H2A.Z	H3K9ac	H3K9ac	H2A.Z
10	H4K20me1	H4K20me1	H4K20me1	H4K20me1	H4K20me1	H2A.Z	H3K9ac
11	H3K27ac	H3K27ac	H3K27ac	H3K27ac	H3K27ac	H3K27ac	H4K20me1

Order	None	MAFF	ZNF263	BDP1	E2F6	RBBP5
1	SMARCB1	MAFF	ZNF263	BDP1	E2F6	RBBP5
2	PML	SMARCB1	SMARCB1	SMARCB1	SMARCB1	PML
3	STAT5A	PML	PML	PML	PML	SMARCB1
4	CTCF	CTCF	STAT5A	STAT5A	CTCF	STAT5A
5	BRF2	STAT5A	CTCF	CTCF	STAT5A	CTCF
6	MAFF	BRF2	BRF2	BRF2	BRF2	BRF2
7	ZNF263	ZNF263	MAFF	MAFF	MAFF	MAFF
8	BDP1	BDP1	BDP1	ZNF263	ZNF263	ZNF263
9	E2F6	E2F6	E2F6	E2F6	BDP1	BDP1
10	RBBP5	RBBP5	RBBP5	RBBP5	RBBP5	E2F6

Table 2: **Performing SSA after one assay has already been performed.** We ran SSA while restricting the output panel to include each assay type in turn, by initializing the submodular greedy algorithm with the assay type in question and then running the algorithm as normal. As expected, restricting the panel to include a particular assay type de-prioritizes assay types that measure similar types of activity. (Top) Choice order of histone modification assays by SSA if one assay type is required to be included. (Bottom) Choice order of transcription factors by SSA if one assay type is required to be included. In both tables, each assay type in the table is encoded with a different color to allow easy comparison of different columns.

Order	$\alpha = 0$	$\alpha = 0.5$	$\alpha = 1$	$\alpha = 5$	$\alpha = 10$
1	NFATC1	NFATC1	NFATC1	NFATC1	H2A.Z
2	ILF2	ILF2	ILF2	H3K4me3	H3K4me3
3	PHF8	PHF8	H3K4me3	H3K9me3	H3K79me2
4	POU5F1	POU5F1	POU5F1	H3K79me2	H3K9me3
5	SMARCC1	SMARCC1	SMARCC1	H3K27me3	H3K27me3
6	IRF4	IRF4	PML	H3K36me3	H3K36me3
7	HNF4G	HNF4G	H3K79me2	H3K4me1	H3K4me2
8	HMGN3	HMGN3	MBD4	H2A.Z	H3K4me1
9	ILF3	H3K4me3	H3K27me3	H3K4me2	H3K9ac
10	ZNF217	H3K79me2	HMGN3	H3K9ac	H4K20me1
11	CCNT2	ILF3	H3K36me3	H4K20me1	H3K27ac
12	PML	ZNF217	H3K9me3	H3K27ac	NFATC1
13	CEBPZ	CCNT2	H3K4me1	ILF2	ILF2
14	NR2F2	PML	IRF4	POU5F1	POU5F1
15	MLL5	CEBPZ	H3K4me2	PML	PML
16	SREBF2	NR2F2	H3K9ac	SMARCC1	SMARCC1
17	XRCC4	MLL5	H2A.Z	MBD4	MBD4
18	IKZF1	SREBF2	ZNF217	HMGN3	HMGN3
19	MBD4	XRCC4	H4K20me1	IRF4	IRF4
20	PRDM1	IKZF1	CCNT2	ZNF217	ZNF217

Table 3: **Incorporating modular weighting to encourage certain assay types to be chosen.** We ran SSA with a variant that weights certain assay types more highly in the selection process. In this experiment, we examine the choice order of assay types by changing the weights on histone modification assays in a selection experiment involving both histone modification and transcription factor assays. We implement this experiment by first defining a combined objective $h^\alpha(A) = f(A) + \alpha m(A)$, where f is the facility location objective and m is the modular function, with each modular score capturing the importance of the item. Note that a set function m is modular if $m(A) = \sum_{a \in A} m(a)$ holds for all $A \subseteq V$. We define a hyperparameter $\alpha > 0$ that controls the trade-off between f and m. We define the modular function m by assigning the score $m(a)$ for each histone mod assay as 1 and for other assay types as 0. Given a choice of α, we use the greedy algorithm to optimize h^α leading to an ordering of the assays. The table displays the resulting order for various choices of α. All histone modification assay types are colored for ease of comparison.

Order	Cell types	Description	Singleton value	Objective gain
1	HBMEC	brain microvascular endothelial cells (mesoderm blood vessel)	48.31	48.31
2	LNCaP	prostate adenocarcinoma (endoderm prostate cancer)	46.77	1.08
3	GM12864	B-lymphocyte (mesoderm blood)	43.74	0.39
4	IMR90	fetal lung fibroblasts (endoderm lung)	43.26	0.37
5	H7-hESC	undifferentiated embryonic stem cells	40.13	0.33

Table 4: The choice order of cell types using SSA methodology.

	Assay Type
1	DnaseSeq
2	H3K4me3
3	CTCF
4	FaireSeq
5	POLR2A
6	H3K27me3
7	H3K36me3
8	H3K79me2
9	H3K4me2
10	H3K9me3

Table 5: The 10 most frequently performed assay types.

Choice order	Assay type	Singleton score	Objective gain
1	H3K4me2	8.12	8.12
2	DnaseSeq	7.66	1.98
3	EZH2	5.19	1.31
4	POLR2A	6.50	1.00
5	NRF1	4.08	0.85
6	JUND	4.64	0.84
7	RAD21	3.74	0.84
8	H3K9me3	2.99	0.84
9	MAX	4.20	0.81
10	H4K20me1	4.81	0.81
11	MXI1	4.45	0.80
12	CEBPB	5.40	0.77
13	USF2	5.83	0.75
14	FaireSeq	5.98	0.75
15	TBP	5.67	0.75

Table 6: The top 15 choice order of SSA on common assay types shared among the five ENCODE tier 1+2 cell types: K562, GM12878, H1-hESC, HepG2, and HeLa-S3.
